# The Impact of Metabolic Scion–Rootstock Interactions in Different Grapevine Tissues and Phloem Exudates

**DOI:** 10.3390/metabo11060349

**Published:** 2021-05-30

**Authors:** Sara Tedesco, Alexander Erban, Saurabh Gupta, Joachim Kopka, Pedro Fevereiro, Friedrich Kragler, Ana Pina

**Affiliations:** 1 Plant Cell Biotechnology Laboratory, Instituto de Tecnologia Química e Biológica António Xavier (Green-It Unit), Avenida da República, Estação Agronómica Nacional, 2780-157 Oeiras, Portugal; Sara.tedesco@itqb.unl.pt (S.T.); psalema@itqb.unl.pt (P.F.); 2 Department 2, Max Planck Institut für Molekulare Pflanzenphysiologie, Wissenschaftspark Golm, Am Mühlenberg 1, 14476 Potsdam-Golm, Germany; Gupta@mpimp-golm.mpg.de (S.G.); Kragler@mpimp-golm.mpg.de (F.K.); 3 Applied Metabolome Analysis Laboratory, Max Planck Institut für Molekulare Pflanzenphysiologie, Wissenschaftspark Golm, Am Mühlenberg 1, 14476 Potsdam-Golm, Germany; Erban@mpimp-golm.mpg.de (A.E.); Kopka@mpimp-golm.mpg.de (J.K.); 4 InnovPlantProtect CoLab, Estrada de Gil Vaz Apartado 72, 7351-901 Elvas, Portugal; 5 Unidad de Hortofruticultura, Centro de Investigación y Tecnología Agroalimentaria de Aragón (CITA), Avenida Montañana 930, 50059 Zaragoza, Spain; 6 Instituto Agroalimentario de Aragón-IA2 (CITA-Universidad de Zaragoza), 50013 Zaragoza, Spain

**Keywords:** grafting, grapevine, metabolic profiles, rootstocks, phloem exudate, scion–rootstock interactions

## Abstract

In viticulture, grafting is used to propagate Phylloxera-susceptible European grapevines, thereby using resistant American rootstocks. Although scion–rootstock reciprocal signaling is essential for the formation of a proper vascular union and for coordinated growth, our knowledge of graft partner interactions is very limited. In order to elucidate the scale and the content of scion–rootstock metabolic interactions, we profiled the metabolome of eleven graft combination in leaves, stems, and phloem exudate from both above and below the graft union 5–6 months after grafting. We compared the metabolome of scions vs. rootstocks of homografts vs. heterografts and investigated the reciprocal effect of the rootstock on the scion metabolome. This approach revealed that (1) grafting has a minor impact on the metabolome of grafted grapevines when tissues and genotypes were compared, (2) heterografting affects rootstocks more than scions, (3) the presence of a heterologous grafting partner increases defense-related compounds in both scion and rootstocks in shorter and longer distances from the graft, and (4) leaves were revealed as the best tissue to search for grafting-related metabolic markers. These results will provide a valuable metabolomics resource for scion–rootstock interaction studies and will facilitate future efforts on the identification of metabolic markers for important agronomic traits in grafted grapevines.

## 1. Introduction

Grafting is an ancient well-established method for plant propagation and improvement. Its discovery likely arose from the attempts of the first agriculturalist for mimicking natural grafting, which allowed the domestication and diffusion of temperate fruit trees [1]. Since then the use of grafting evolved from being merely a means of propagation towards its use to improve resilience against biotic and abiotic impacts [2] and has become a common method used not only in orchards and viticulture but also in horticulture and ornamentals. A prominent example is found with grapevines and the spread of Phylloxera in Europe since the middle of the 19th century. Grafting *V. vinifera* scions onto Phylloxera-resistant American rootstocks represents the longest use of a biological control strategy that avoids expensive and elaborate quarantine controls [3].

The use of grafted plants has many agronomical advantages. For instance, grafting is particularly useful for reducing the period of juvenility in perennial plants [4]. The ability of dwarfing rootstocks in reducing scion vigor is widely exploited in commercial fruit production [5]. Grafting also improves plant growth under environmental stresses, such as drought [6,7] and salt stress [8,9]. In addition, the effects of rootstock–scion interaction on growth, fruit quality, and stress tolerance have been widely reviewed [10,11]. Therefore, understanding scion–rootstock interactions is crucial for choosing the most suitable graft combinations for specific environments and good fruit quality [10]. Nevertheless, grafting also constitutes a source for pathogen dissemination given that, by grafting fungal, bacterial, and viral biomes of grafted plants interact and might have a role in the healing of the graft union and the final performance of the plant. Despite this, grafting, when implemented carefully, has greatly contributed to the intensification of agriculture.

The effects of grafting, which produces a chimeric organism, are complex and currently largely unpredictable [12]. Chimeric plants produced by grafting have been used to study long-distance movement of signaling molecules, especially via phloem, such as sugars, hormones, proteins, silencing inducing RNAs, and messenger RNAs [13,14,15]. The identification of the mobile transcription factor, FLOWERING LOCUS T (FT), as the putative “florigen” thought to be the key for the transition to flowering was a major achievement in the past decades and was uncovered by grafting, as FT is produced in leaves but translocated to the shoot apex to exert its function [16]. Crosstalk between the above and below graft parts is conducted by plant vascular systems, xylem, and phloem [17]. While xylem sap is easy to collect, considerable obstacles to access the phloem content lies in the fact that the phloem seals itself upon wounding. However, the phloem exudates from stems, petioles, or floral axes incisions can be collected with the use of chelating agents, such as EDTA, to eliminate sieve tube blockage. It is a well-established method that allowed the unveiling of phloem content and its dynamics in many plant species [18,19]. Nevertheless, it must be taken into account that only relative quantification of the phloem sap can be performed since it is an exudation rather than a direct collection of the phloem sap [18]. New omics approaches have recently been applied in grafting studies to dissect the molecular mechanisms of the early graft-junction formation [20], to unveil the phenomenon of graft compatibility [21,22,23], and to understand the scion–rootstock interactions leading to the alteration of agronomically important traits [24,25,26]. Metabolites, as the end-product of gene expression and regulation, have also been investigated in grafted grapevine [27] and citrus trees [17] and were associated to graft formation and fruit quality. Viruses, phenolic compounds, and flavonoids have been proposed as markers for graft incompatibility in *Vitis* [28,29] and *Prunus* [30,31] and secondary metabolism appears to be increased in heterografted grapevines when compared to homografts (i.e., a graft between two individuals of the same genotype) [32]. Indeed, graft success depends not only on the genotype of each plant part and the grafting protocol used to combine the scion and rootstock but also on the reciprocal signals transmitted between these two plant body parts [2]. However, to date, we have a limited understanding of the signals exchanged between scion and rootstock. Recently, it was shown that grapevine scion–rootstock interactions affect important developmental decisions and growth habits of the scion just 5 months after grafting, at the time when the healing of the graft is not yet complete [33]. In order to shed light on the early metabolic grapevine scion–rootstock interactions between the grafting partners, we investigated changes in the global metabolic profiles in eleven homograft and heterograft grapevine combinations in leaves, stem, and phloem exudates that were collected from both above and below the graft union at 5–6 months after grafting. In particular, we assessed (1) the metabolic profile of homografts and heterografts, (2) the effect of a heterologous grafting partner in the metabolome of a plant in specific tissues and phloem exudates samples, and (3) the metabolic profile of scion and rootstock samples.

## 2. Results

Given the impact of the tissue in the distribution of the data (Supplementary Figure S1), the analyses of homografts vs. heterografts and of paired comparisons in phloem and stems dataset were carried out separately for scion and rootstock samples.

### 2.1. Metabolic Profile of Homografted and Heterografted Grapevines in Tissues and Phloem Exudates Collected from the Scion and Rootstock

**Figure 1:**
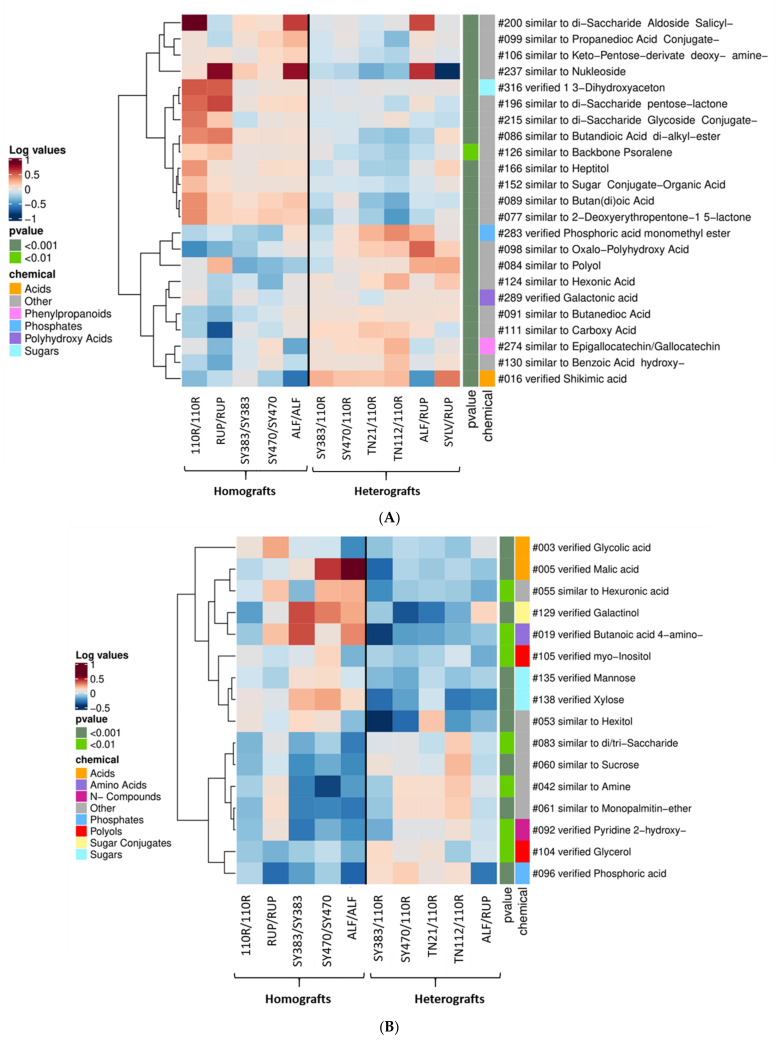
Heat map clustering analysis of homografts versus heterografts at 5–6 months after grafting: (**A**) In the **leaves**; (**B**) and the **rootstock phloem exudate**’ datasets. Leaves and phloem rootstocks’ metabolites found to be increased or decreased in at least 80% of the homograft combinations and less than 20% of the heterografts (i.e., n = 23 and 16, respectively). Selected metabolites were retrieved from all metabolites found significantly different in at least one of the leaves and phloem rootstock paired-comparison of a homograft versus a heterograft at *p* < 0.05 according to the Tukey post hoc test (i.e., n = 231 and 30, respectively). Mean log_10_-transformed values per graft combination are plotted, as well as the *p*-value range (<0.01 and <0.001) and the potential chemical class (“chemical”) of plotted metabolites. Not-verified metabolites (named with the prefix “similar to”) were included in the chemical class assigned as “Other”. Cluster analysis of metabolites was performed using the Pearson correlation method.

The different metabolic profiles in the scion and rootstock tissues and phloem exudates of homografts and heterografts were analyzed for their significant reciprocal changes (95% confidence level). In **leaves**, 23 metabolites (Figure 1A) were found consistently changed between homografts and heterografts. When homografts were compared to heterografts, several sugars were significantly increased, such as 1,3-dihydroxyaceton; and several other not-verified compounds traceable as disaccharides, carbohydrates, and sugar conjugates (Figure 1A). Aside from carbohydrates, a few compounds related to carboxylic acids, such as a conjugate of propanedioic acid, butan(di)oic acid, butandioic acid di-alkyl-ester, and carboxylic esters (2-deoxyerythropentone-1,5-lactone), were also found significantly increased in homografts. In contrast, among the metabolites that are significantly increased in heterografts comparing to homografts, we detected phosphoric acid monomethyl ester, galactonic acids, and shikimic acids and other not-verified acid compounds such as carboxylic acids, like butanedioic acid among others. In heterografts, phenolic compounds similar to epigallocatechin or gallocatechin and benzoic acid hydroxy, a phenolic acid derived from the phenylpropanoid pathway [34], increased compared to homografts alongside the already mentioned shikimic acid, a central metabolite for the regulation of phenolic metabolism [35].

In the **scion phloem exudate** of homografts and heterografts, just one metabolite, the sugar alcohol threitol, was found consistently more abundant in the phloem exudate of homografts (0.09 ± 0.053 SE) than in heterografts (−0.2 ± 0.1 SE; Tukey post hoc test at *p* < 0.05). However, 16 metabolites consistently differed in homografts when compared to heterografts in the **rootstock phloem exudate**. Figure 1B shows that the amino acid 4-amino butanoic acid (GABA), considered an important signal molecule, is consistently more abundant in the phloem exudate of homografts rather than of heterografts together with glycolic and malic acids. Furthermore, sugars, such as mannose and xylose, the sugar conjugate galactinol, and the polyols (sugar alcohols) *myo*-inositol were all increased more in homografts than in heterografts. On the contrary, the metabolites that appeared more abundant in the heterografted combinations were the N-compound 2-hydroxy-pyridine, phosphoric acid, and the polyol glycerol.

**Figure 2:**
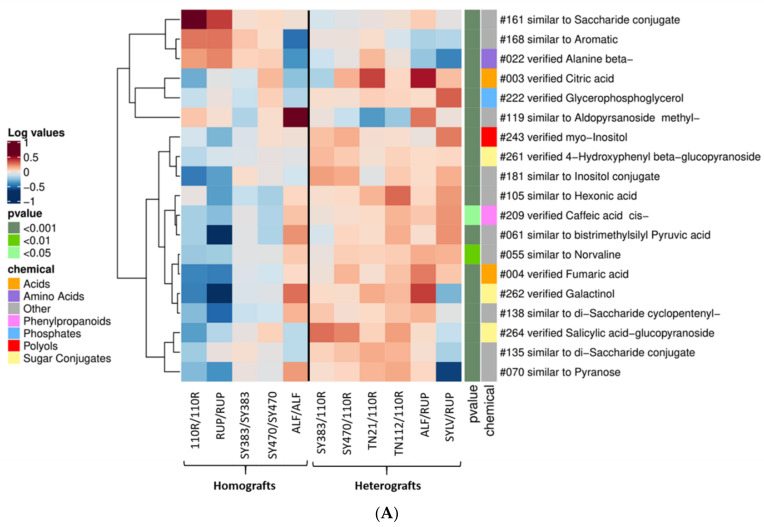

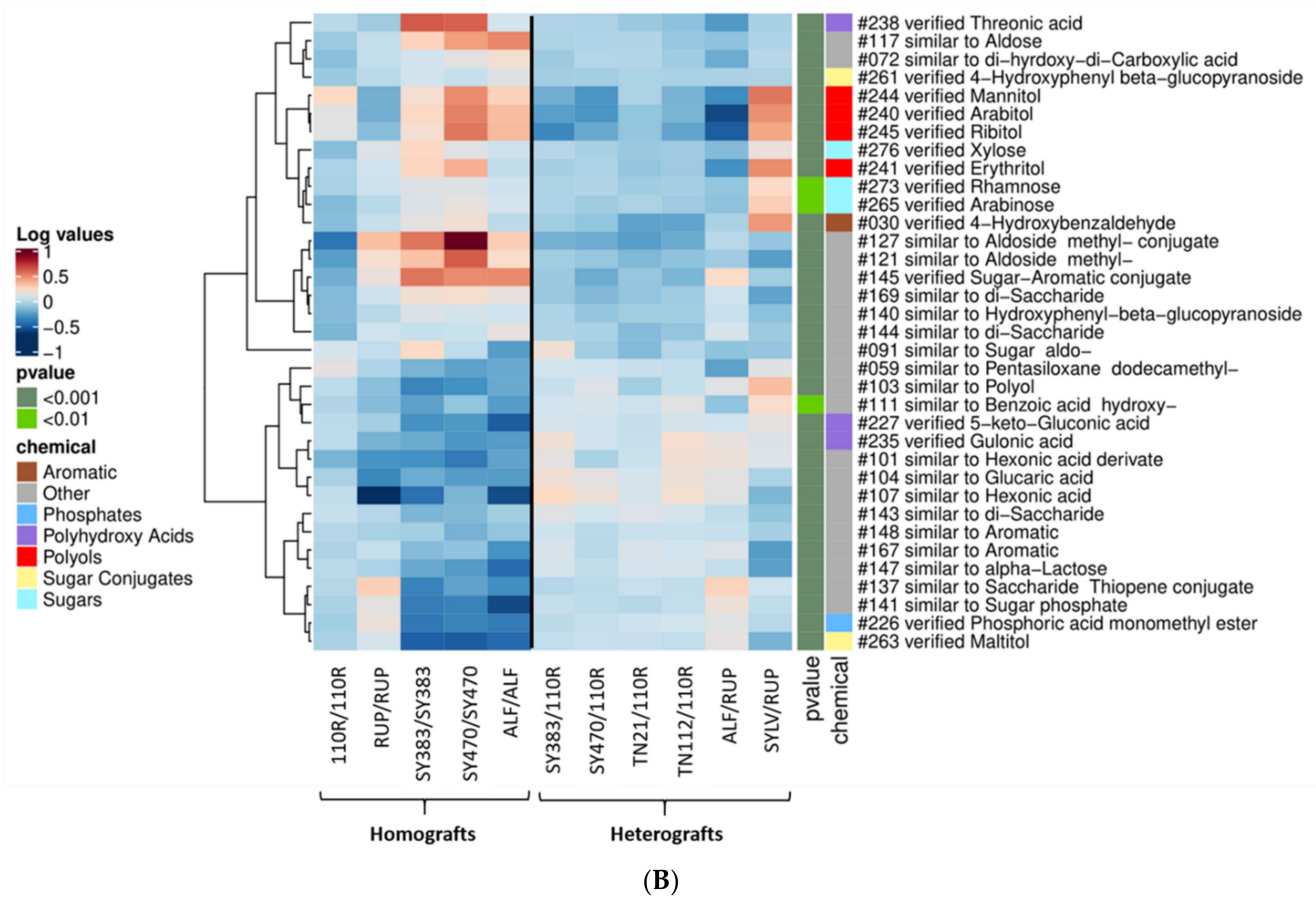
Heat map clustering analysis of homografts versus heterografts at 5–6 months after grafting: (**A**) in the **scion stems**; (**B**) the **rootstock stems**’ datasets. Scion and rootstock stems’ metabolites found increased or decreased in at least 80% of the homograft combinations and less than 20% of the heterografts (i.e., n = 19 and 35, respectively). Selected metabolites were retrieved from all metabolites found significantly different in at least 1 scion and rootstock stem paired-comparison of a homograft versus a heterograft at *p* < 0.05 according to Tukey post hoc test (i.e., n = 157 and 159, respectively). Mean log_10_-transformed values per graft combination are plotted, as well as the *p*-value range (<0.05, <0.01, and <0.001) and the potential chemical class (“chemical”) of plotted metabolites. Not-verified metabolites (named with the prefix “similar to”) were included in the chemical class assigned as “Other”. Cluster analysis of metabolites was performed using the Pearson correlation method.

Regarding the metabolic differences found in the **scion stems**, 19 metabolites were consistently different in homografts versus heterografts (Figure 2A).

A higher number of metabolites was increased in heterografts vs. homografts, 16 and 3, respectively. Among these, citric acid, glycerophosphoglycerol, *myo*-inositol, 4-hydroxyphenyl beta-glucopyranoside, cis-caffeic acid, fumaric acid, galactinol, and salicylic acid-glucopyranoside were all verified (Figure 2A). In addition, 35 metabolites were consistently different between the two groups in the **rootstock stems**. Figure 2B shows that several polyols such as mannitol, arabitol, ribitol, and erythritol; several sugars such as xylose, rhamnose, and arabinose; as well as the sugar conjugate 4-hydroxyphenyl beta-glucopyranoside were all found increased in homografts when compared to heterografts together with threonic acid, 4-hydroxybenzaldehyde, a sugar-aromatic conjugate, and other not-verified compounds. Conversely, polyhydroxy acids such as 5-keto-gluconic acid and gulonic acid, the sugar conjugate maltitol, and phosphoric acid monomethyl ester were increased in heterografts when compared to homografts. Moreover, several not-verified compounds, especially substances attributable as acids, aromatics, and polyols were found to be increased in heterografts when compared to homografts in the rootstock stems (Figure 2B).

### 2.2. Grafting Partner Induced Changes in the Scion and Rootstock Metabolome

**Figure 3:**
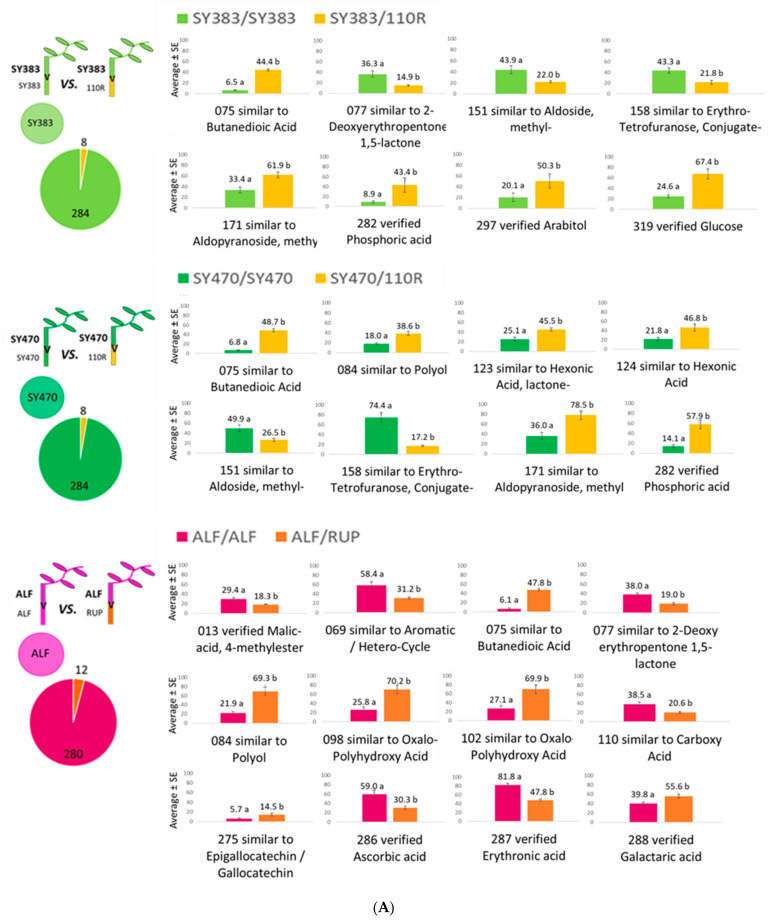

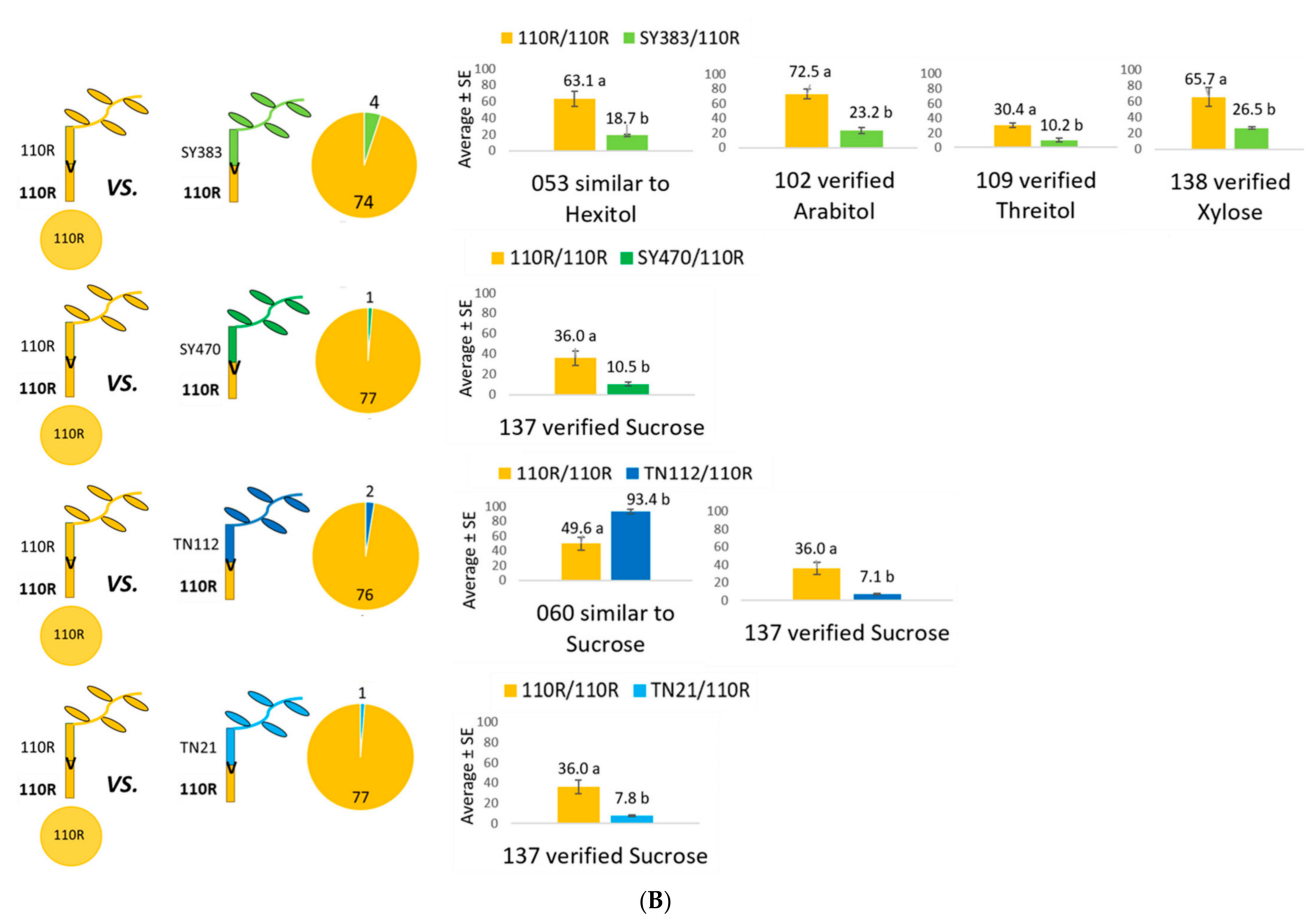
Scale of the effect and significant different metabolites: (**A**) in the **leaves**; (**B**) the **rootstock phloem exudate**’ samples of homografts upon grafting with a heterologous partner. Scale of the effect of a heterologous grafting partner in the leaves and phloem rootstocks’ metabolome of a homograft and pie graphs with the number of changed and unchanged metabolites (left); bar charts of the mean value of significant different metabolites upon grafting with a heterologous partner (right). The different letters indicate significant differences between the graft combinations at *p* < 0.05 according to the Tukey post hoc test. Data are presented as the average of data normalized to the maximum value for each metabolite. Bars represent the standard error.

In order to elucidate how a heterologous grafting partner affects the metabolic composition of the other grafting partner, we compared each homograft tissue (and phloem exudate) with the same tissue (and phloem exudate) of its respective heterograft. In the **leaves** dataset, 8 of 292 identified metabolites were different in homografted SY383 and SY470 when compared with their respective heterografts SY383/110R and SY470/110R and 12 of 292 identified metabolites were found to be different between the leaves of ALF/ALF compared to ALF/RUP at *p* < 0.05 according to the Tukey post hoc test (Figure 3A).

The 110R and RUP rootstocks seem to induce a compound similar to butanedioic acid in the leaves of *V. vinifera* cv. Syrah clone 383 and 470 and of cv. Alfrocheiro than when self-grafted. Phosphoric acid and a compound attributable to aldo-pyranoside methyl- were increased while compounds similar to aldoside methyl and erythrotetrafuranose conjugates were reduced in the leaves of both Syrah clones in response to the presence of 110R rootstock. A polyol compound was detected to be increased in SY470 and ALF leaves that are grafted onto 110R and RUP, respectively, rather than when in self-grafted plants. Similarly, a compound traceable as 2-deoxyerythropentone-1,5-lactone was found to be increased in homografts of SY383 and ALF in response to the 110R and RUP rootstocks, respectively. Some metabolites were differently affected by both rootstocks in a genotype-specific manner. For instance, 110R leads to an increased content of arabitol and glucose in the leaves of SY383/110R compared to SY383/SY383 but not compared to SY470/110R, where 110R induced an increase in substances similar to hexonic acids and hexonic acid lactone (compared to the leaves of SY470 homografts) (Figure 3A).

Interestingly, the **rootstock phloem exudate** showed more metabolic changes in the presence of a heterologous scion than the phloem exudate from the scion (data not shown) in the presence of a heterologous rootstock, according to the source-to-sink (scion and rootstock, respectively) phloem flow. Indeed, when the rootstock phloem exudate of 110R homografts were compared with respective heterografted SY383, SY470, TN112, and TN21 scions, out of the 78 identified phloem exudate metabolites 4, 1, 2, and 1 metabolites were displayed as significantly different, respectively (Figure 3B). Nevertheless, no metabolite was significantly different when compared between the phloem exudate of RUP/RUP and the respective heterografted ALF/RUP exudate. It is worth it to point out that sucrose appeared significantly reduced in the phloem exudates of heterografted 110R rootstocks when compared to the self-grafted 110R/110R exudate, except for SY383/110R. SY383/110R phloem exudate showed reduced xylose and polyols (namely threitol, arabitol, and a compound attributable to hexitol) amounts in comparison to 110R/110R exudate. There was only one not-verified metabolite similar to sucrose found to be increased in TN112/110R phloem exudate with respect to 110R/110R, which might hypothetically compensate for the sucrose depletion seen in heterografts with 110R rootstock (again with SY383/110R being an exception) (Figure 3B).

**Figure 4:**
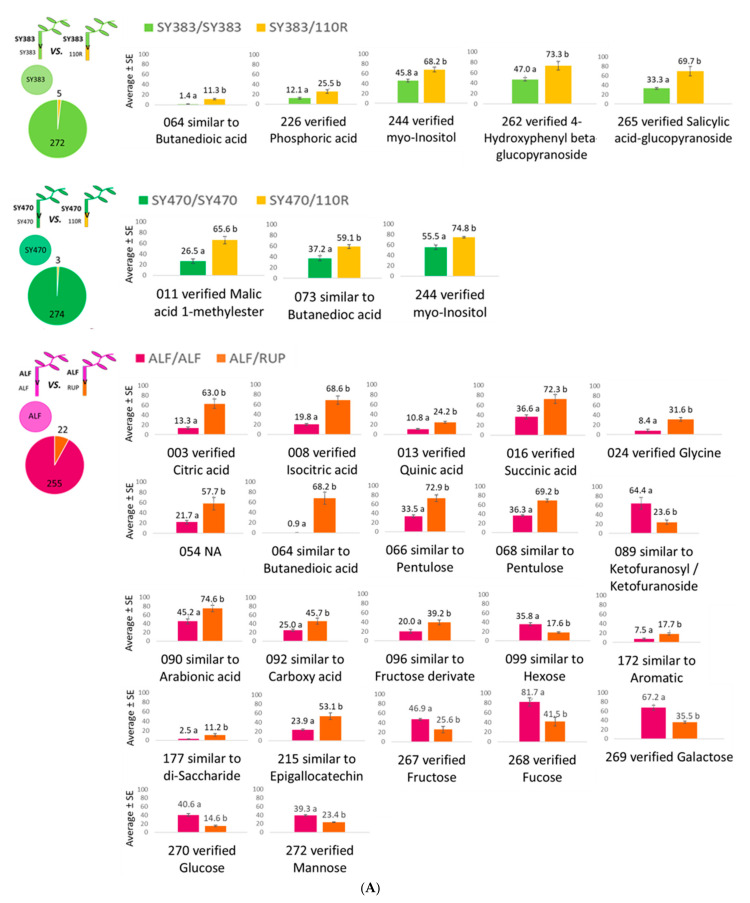

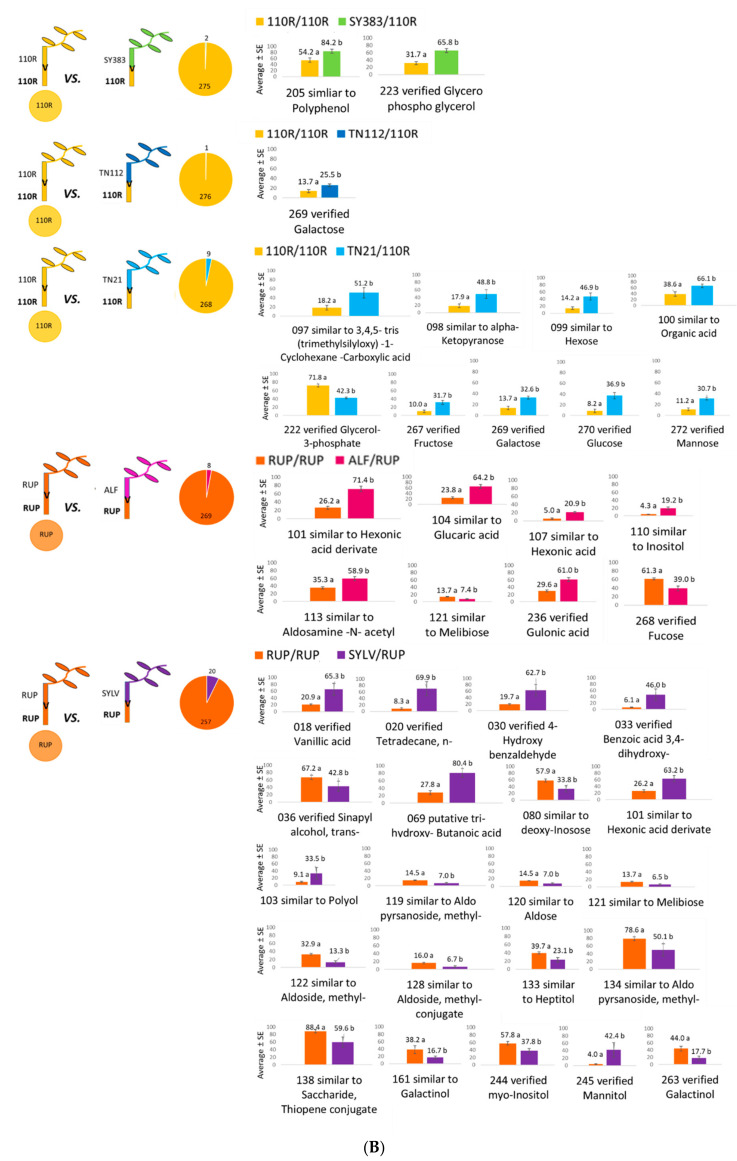
Scale of the effect and different significant metabolites: (**A**) in the **scion stems**; (**B**) the **rootstock stems** of homografts, upon grafting with a heterologous partner. Scale of the effect of a heterologous grafting partner in the scion and rootstock stems’ metabolome of a homograft and pie graphs with the number of changed and unchanged metabolites (left); bar charts of the mean value of significant different metabolites upon grafting with a heterologous partner (right). Different letters indicate significant differences between the graft combinations at *p* < 0.05 according to the Tukey post-hoc test. Data are presented as the average of data normalized to the maximum value for each metabolite. Bars represent the standard error.

Concerning the effect of rootstocks on **scion stems**, among the 277 identified metabolites only 5 metabolites were found different between the homografted SY383/SY383 and heterografted SY383/110R, 3 metabolites between SY470/SY470 and heterografted SY470/110R, and 22 metabolites were found different in ALF/ALF when compared to the same tissue of ALF/RUP at *p* < 0.05 according to the Tukey post hoc test (Figure 4A).

As seen in Figure 4A, only not-verified compounds similar to butanedioic acid were found increased in all heterograft combinations (SY383/110R, SY470/110R, and ALF/RUP) when compared to their respective homografts. Similarly, *myo*-inositol was also increased in SY383/110R and SY470/110R heterografts compared to their respective homografts, while phosphoric acid, the phenolic glycoside 4-hydroxyphenyl beta-glucopyranoside, and salicylic acid-glucopyranoside were specifically found increased in SY383/110R with respect to SY383/SY383. Conversely, malic acid 1-methylester was enriched in SY470/110R when compared to SY470/SY470. Several metabolites were specifically altered in ALF stem grafted onto RUP rootstock. For instance, when comparing ALF/ALF to ALF/RUP, several acids such as citric, isocitric, quinic, and succinic acids; as well as several other not-identified compounds including the amino acid glycine and a phenolic similar to epigallocathechin/gallocathechin were increased in ALF/RUP. On the other hand, several sugars such as fructose, fucose, galactose, glucose, and mannose were found depleted in the scion stems of the heterograft ALF/RUP when compared to ALF/ALF (Figure 4A). Regarding the effect of a heterologous scion on the metabolome of **rootstock stems** (Figure 4B), this was higher in the RUP rootstock than in 110R depending on the scion used. Only two metabolites were found different between the rootstock stem of 110R/110R and SY383/110R, no differences were found in SY470/110R rootstock stem, and one and nine metabolites were found different between 110R/110R homografts and TN112/110R and TN21/110R heterografts, respectively.

However, 8 and 20 metabolites changed when RUP was grafted with ALF or SYLV scions, respectively at *p* < 0.05 according to Tukey post hoc test (Figure 4B). The phosphate glycerophosphoglycerol and a substance attributable to a polyphenol increased in SY383/110R relative to the 110R /110R rootstock stem. The sugar galactose was more abundant in the 110R rootstock with TN21 and TN112 scions compared to 110R/110R. Interestingly, TN21 as a scion also induced other metabolic changes in the 110R rootstock stem. In addition to galactose, other sugars (i.e., fructose, glucose, and mannose) were increased in TN21/110R when compared to 110R/110R. In these samples, other not-verified compounds were also found to be increased, while only glycerol-3-phosphate was reduced in TN21/110R when compared to 110R/110R. Changes in RUP rootstock stems were affected in a genotypic-specific manner as only two metabolites, namely compounds traceable as a derivate of hexonic acid and melibiose, were found to be commonly altered by the presence of ALF and SYLV scions. As mentioned, SYLV scion caused more metabolic changes in the rootstock stems of RUP than an ALF scion (Figure 4B). In addition to the sugar conjugate galactinol, the polyol *myo*-inositol, and *trans*-sinapyl alcohol, which were found depleted in the heterograft SYLV/RUP, all the other verified compounds, namely the polyol mannitol, vanilic acid, the aliphatic tetradecane, n-, the aromatics 4-hydroxybenzaldehyde, and benzoic acid 3,4-dihydroxy-, were found increased in the rootstock stems of SYLV/RUP when compared to RUP/RUP.

### 2.3. Metabolic Profiles of Scion and Rootstock Phloem Exudates and Stems in Grafted Grapevines

**Figure 5:**
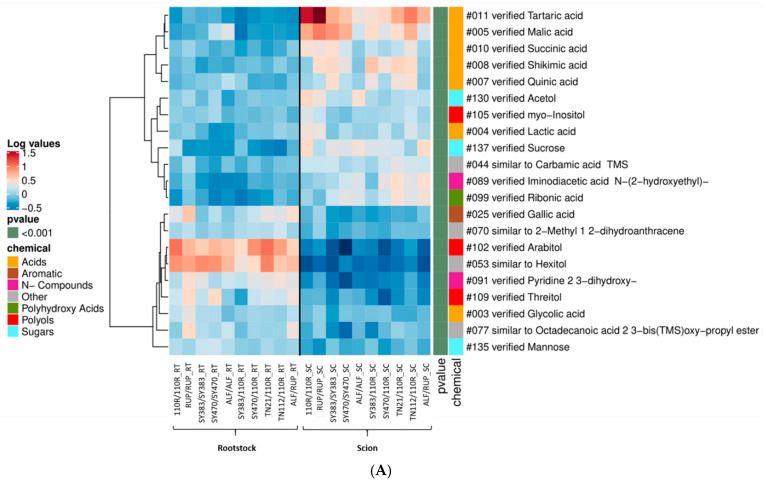

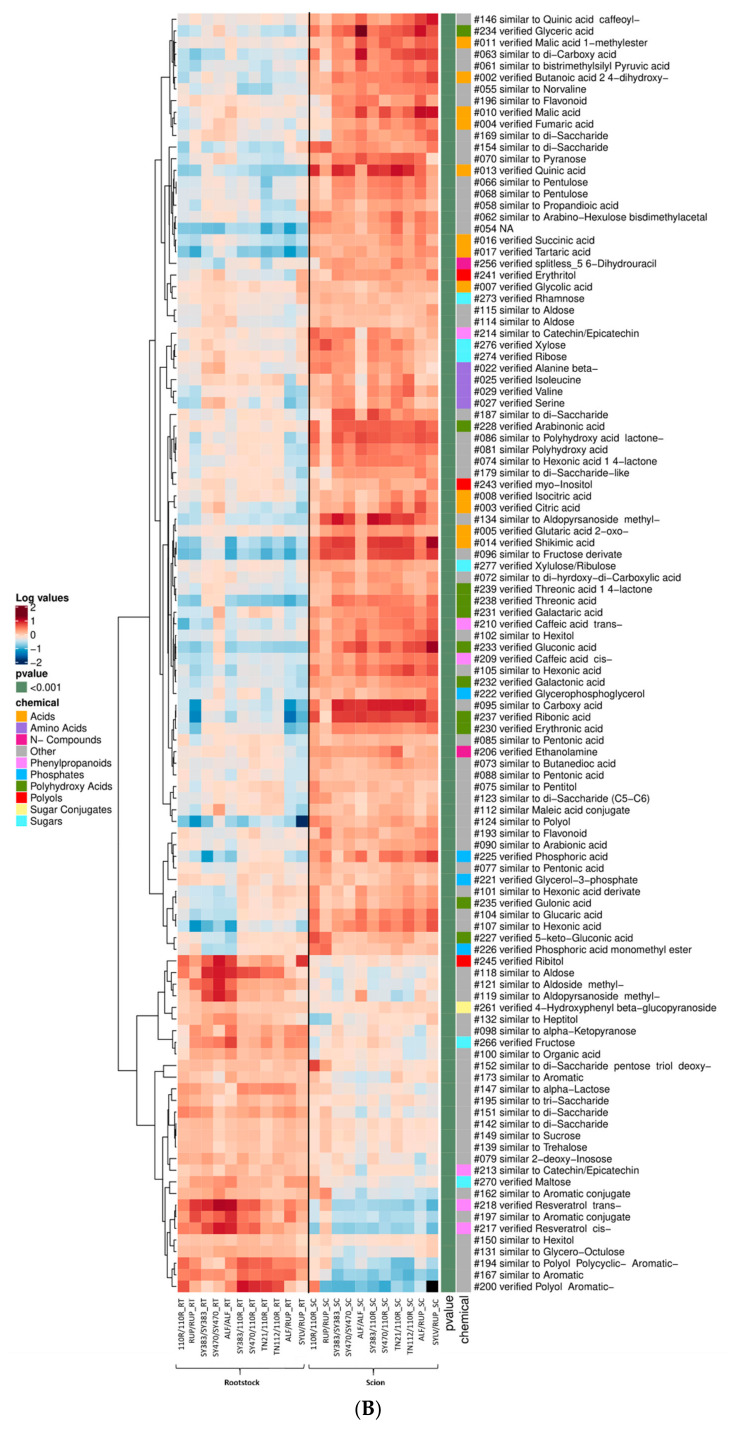
Heatmap clustering analysis of scion vs. rootstock at 5–6 months after grafting: (**A**) in the **phloem exudate**; (**B**) **stems** datasets. Phloem exudate and stems metabolites found increased or decreased in at least 80% of the scion and less than 20% of the rootstock samples. Selected metabolites were retrieved from all metabolites found significantly different in at least one paired-comparison of a scion vs. a rootstock sample at *p* < 0.05 according to the Tukey post hoc test. Mean log_10_-transformed values per graft combination are plotted (missing values are visualized in black color), as well as the *p*-value range (<0.001) and the potential chemical class (“chemical”) of plotted metabolites. Not-verified metabolites (named with the prefix “similar to”) were included in the chemical class assigned as “Other”. Cluster analysis of metabolites was performed using the Pearson correlation method.

To better investigate the scion–rootstock cross-talk and to shed light on the huge impact imposed by the tissue (i.e., scion or rootstock) on the metabolic profiles analyzed (highlighted by the PCAs shown in Figure S1), we compared phloem exudate and stem samples collected from above (scion) and below (rootstock) the graft union. Following the same criterium used for the previous heat maps, significant metabolites with an inverted and consistent behavior among the graft combinations of the scion (_SC) and rootstock (_RT) samples were included in Figure 5. Concerning the composition of the **phloem exudate**, 21 metabolites were consistently found different in samples collected above than in the ones collected below the graft union (Figure 5A).

Figure 5A shows that several acids (i.e., tartaric, malic, succinic, shikimic, quinic, lactic, and ribonic acids) were found increased in the phloem exudate collected from the scion compared to the rootstock. Sucrose, the polyol *myo*-inositol, acetol, and a N-compound (iminodiacetic acid N-(2-hydroxyethyl)) were also found increased in phloem exuded from scions than from rootstocks. Conversely, the sugar mannose and the polyols, threitol and arabitol, were reduced in the phloem from scion than from rootstock. The phenolic compound gallic acid, the N-compound 2,3-dihydroxy-pyridine, and glycolic acid were also found increased in phloem harvested from the rootstock than from the scion (Figure 5A).

In **stems**, 111 metabolites were detected to consistently differ among the graft combinations in samples collected from the scion (_SC) and the rootstock (_RT). Many metabolites seem to be increased in the stems collected from the scion rather than from the rootstock (i.e., 82 and 29 metabolites, respectively) as displayed in Figure 5B. Considering the verified metabolites found enriched in the scion stems when compared to the rootstock stems, we found several acid compounds namely malic acid 1 -methylester, butanoic acid 2,4-dihydroxy-, malic, fumaric, quinic, succinic, tartaric, glycolic, citric, isocitric, shikimic acids, and glutaric acid 2-oxo-. Moreoever, polyhydroxy acids such as glyceric, arabinonic, threonic and threonic acid 1,4-lactone, galactaric, gluconic, galactonic, ribonic, erythronic, gulonic acids, and 5-keto-gluconic acid increased in the scion stems rather than in the rootstock stems together with the amino acids alanine beta-, isoleucine, valine, and serine. A few phosphates, namely glycerophosphoglycerol, phosphoric acid, glycerol-3-phosphate, and phosphoric acid monomethyl ester; the phenols, *cis*- and *trans*-caffeic acids; the polyols erythritol and *myo*-inositol; the sugars rhamnose, xylose, ribose, and xylulose/ribulose; the N-compound 5,6-dihydrouracil; and ethanolamine were all found more abundant in the stems collected from the scion than from the rootstock. Other than these, many other not-verified compounds mainly traceable as acids, polyhydroxy acids, and several sugars and their conjugates were higher in scion stems than in rootstocks (Figure 5B). Interestingly, compounds similar to flavonoids and phenolics traceable as catechin/epicatechin, caffeoyl-quinic acid, and the already mentioned *cis*- and *trans*-caffeic acid were more abundant in the scion than in the rootstock stems. Conversely, only a few metabolites were displayed as enriched in rootstock stems when compared to scion stems. Among these, we can find the sugars fructose and maltose, the sugar conjugate 4-hydroxyphenyl beta-glucopyranoside, the polyol ribitol, a polyol aromatic-, and the phenolic compounds *cis*- and *trans*-resveratrol. Likewise, not-verified substances that seem to belong to sugars, sugar conjugates, and aromatics and their conjugates, including a phenolic similar to catechin/epicatechin, appear depleted in the scion stems rather than in the rootstock ones (Figure 5B).

## 3. Discussion

To elucidate the metabolite content and the changes resulting from scion–rootstock interactions in nursery-grafted grapevines, we have profiled the metabolome of leaves, stems, and phloem exudate collected from above and below the graft union of 11 graft combinations five to six months after grafting. Results from the PCAs (Figure S1), performed for each of the investigated sample type, indicate that grafting had a minor impact on the metabolome of grapevine when tissues or genotypes were compared. The tissue (e.g., scion stem vs. rootstock stem of the harvested material) is the highest variance factor which is expected considering that scion stems are herbaceous tissues and rootstocks stems are lignified. Interestingly, although the phloem composition is not expected to vary much between grapevines, the phloem exudate deviated more than what was expected.

### 3.1. Heterografting Enhances Defense-Responses in Both Scions and Rootstocks

Concerning the scale of the scion–rootstock interactions in homografts vs. heterografts, rootstocks are more affected by the presence of a selfgrafting or a heterologous grafting partner than scions are. Indeed 13 and 30 significant changes were detected between at least one homograft vs. one heterograft in scion and rootstock phloem exudates, respectively (being 1 and 16 the consistent changed metabolites respectively, on 78 identified metabolites), and 158 and 159 were the metabolic changes detected in scion and rootstock stems, respectively (being 19 and 35 the consistent changes, on 277 identified metabolites). Considering the source-to-sink flow of photoassimilates and that the scion is the photosynthetic producer of the grafted plant, it is not surprising that the rootstock grafting partner, acting as a net sink, was most affected by the presence of a heterologous one. Qualitatively, the profile of homografts vs. heterografts highlighted that sugars are increased in homograft samples both above and below the graft union when compared to heterografts (Figure 1A,B and Figure 2B) suggesting a more active carbo metabolism in the leaves of homografts and a more effective phloem translocation across their graft interfaces. Indeed, sugars and GABA were found increased in the rootstock phloem exudate of homografts when compared to heterografts. Recently, GABA was also enriched at the graft interface of homografted grapevines compared to the tissues of scion and rootstock [27]. As GABA is considered an important signaling molecule, with roles in plant responses to stress and the carbon:nitrogen balance [36], the enrichment in GABA in the rootstock phloem exudate of homografts might indicate an earlier or a stronger response against the stress induced (directly or indirectly) by grafting in homografts rather than in heterografts. An increased content in carboxylic acids, possible intermediaries of the TCA cycle, and an enhanced phenolic metabolism was found increased in scion leaves and the stems of heterografts when compared to homografts, while below the union, heterografted stems were enriched in polyhydroxy acids. To the best of our knowledge, an enrichment in acid compounds in leaves and stem samples from heterografts when compared to homografts (both above and below the union) was not previously reported in grafting studies, particularly the enrichment in carboxylic acids identified in scion stems. For more than 30 years, metabolites such as sugars and acetate are known to repress the promoter activities of selected photosynthetic genes, while nitrate, amino acids, and several carboxylic acids are known to induce their transcription [37]. Evidence that TCA cycle intermediates act in regulating transcript abundances has been collected in humans [38], yeasts [39], and plants [40,41] and they are considered good candidate signaling molecules since they reflect both the metabolic and redox status of a cell and are transported between compartments [41]. Therefore, it is not an excluded consideration that the carboxylic acids detected in heterografted scion stems might play a role in the perception of a foreign partner and the adaptation of its gene expression. Caffeic acid, already proposed as related to pathogen resistance in grapevine [42], culminated with other defense-related compounds were found specifically increased in heterografted stems collected a few centimeters above the graft union. Among these, the phenolic glycoside known as arbutin (hydroxyphenyl beta-glucopyranoside) was identified several times in grapevine pathogenesis studies [43], such as upon colonization by endophytic bacteria [44]. Likewise, a glycoside of salicylic acids, important against biotic threats, and the oligosaccharide galactinol involved in antioxidant protection were more abundant in scion stems of heterografts than in self-grafted grapevines. In leaves, other phenolic compounds and shikimic acid were also reported to be increased in heterografts. Below the union, 2-hydroxy-pyridine, a pyridine-based alkaloid compound known to be induced by stress (especially wounding as feeding deterrent) [45], was increased in the rootstock phloem exudate of heterografted vines. Overall, our study confirms an enhanced phenol metabolism in heterografted grapevines supporting the notion that the presence of a non-selfgrafting partner induces a defense-related response, as previously suggested by comparing the transcriptomes of homografted and heterografted grapevines [32]. In this work, we have shown that the presence of a heterologous scion (Figure 1A and Figure 2A) or rootstock (Figure 1B and Figure 2B), not only leads to a local induction of defense-related compounds but also it is detected in leaf tissue and rootstock phloem exudates. Considering that the highest number of intracellular pathogens ever found in a single crop was recorded in grapevines [46], it would be interesting to verify whether the enhanced stress response imputed to heterografted vines might reflect the perception of a foreign biome and/or the interaction of the grafting partner’s biomes when these belong to different genotypes. Indeed, many of the identified defense-related compounds such as phenols, sugars, and metabolites from the salicylic acid pathway were found altered in virus-infected grapevines [47]. Therefore, it is not an excluded consideration that viruses might have a role in the detection of the heterografting-induced defense response. In this regard, viruses were reported to cause graft incompatibility in grapevines [48], which is understandable given that more than 65 viruses have been recorded to infect grapevines, but just a few of these viruses are tested in the EU certification schemes [49].

### 3.2. Scions and Rootstocks Are Able to Affect Specific Tissues and Phloem Exudates within the Grafted Plant

In the last decades, several lines of research have focused on the rootstock-induced alterations of several important scion agronomical traits and the interest in deliberately altered phenotypes by mean of grafting [2]. In this study, the results highlighted that in grafted grapevines the rootstock is more affected due to the presence of a heterologous partner than the scion when comparing homografts vs. heterografts. However, whether this impact on the metabolome is directly induced by the rootstock genotype or if it can be attributed to more complex consequences of an altered rootstock metabolism per se in response to the scion genotype is unknown. In order to better understand the reciprocal impact of one grafting partner to the other, we compared the metabolome of leaves, stems, and phloem exudate collected from both above and below the graft union of homografts with respective heterografts (Figure 3 and Figure 4). Results showed that the scale of the scion–rootstock reciprocal interaction is relatively small and, at certain times, no metabolite was altered in response to a different grafting partner. Nevertheless, such changes are shown to be differentially driven not only by the specific genotypic graft composition but also by the specific samples, suggesting that scions and rootstocks are able to affect specific organs and phloem exudates within the grafted plant (Figure 3 and Figure 4). For instance, the 110R rootstock phloem metabolome was more affected by the presence of a different scion genotype than its rootstock stem metabolome. In contrast, no metabolomic effect was observed in the phloem exudate when a different scion genotype was grafted onto a RUP rootstock but the effect was higher and genotypically-driven in the rootstock stem metabolomes. It has recently been proposed, based on metabolic changes detected in grafted citrus trees, that an effect of rootstocks on scions might be driven in a distance-dependent manner [17]. However, we found that in grapevines both grafting partners exert their influence in specific organs and phloem exudates independently of their distance but rather depending on the specific graft combination.

Qualitatively, a relative high number of changes were consistently detected in scion leaves that were dependent on the rootstock genotype. Hence, among the investigated samples, leaves seem to be the best tissue to search for grafting-related metabolic markers. As it is shown in Figure 3A and Figure 4A, both American rootstocks (110R and RUP) induced an increase in a compound similar to the carboxylic acid butanedioic acid in scion leaves and stems compared to self-grafted plants. This suggests that this increase is a specific response to the American rootstocks. On ALF scion stems, other carboxylic acid intermediates of the TCA cycle and phenolic compounds were also increased when grafted onto RUP rootstock, while the *myo*-inositol content of both Syrah scion stems was found increased when grafted onto 110R indicating an enhanced defense metabolism of cv. Syrah in response to 110R rootstock. Different grapevine rootstocks were already reported to induce different strategies of defense-related responses in scion leaves and were suspected to be potentially involved in the priming phenomenon, which is a defensive measure in which the plant is in a persistently primed state of enhanced defense readiness [50]. Furthermore, carboxylic acids were suggested to act as priming agents in Arabidopsis under *Pseudomona* infections enhancing gene expression of factors regulating the salicylic and jasmonic acid defense pathways [51]. Related compounds such as 3-hydroxybutanoic acid was proposed as a downy mildew resistance biomarker of grapevine leaves, while isomers of 2,3,4-trihydroxybutanoic acid and *myo*-inositol were related to the susceptibility [52]. Nevertheless, it remains to be shown whether the defense-related responses induced in the scions by the rootstock enhances stress tolerance or if these defense-responses directly respond to the perception of a different grafting partner (or to its biome).

As mentioned, the effect of a scion on the rootstock stem metabolome was stronger in RUP rather than in 110R and the changes were mostly dependent on specific scion-rootstock combinations rather than generalized, as only galactose was found increased in 110R stems due to the effect of both Touriga Nacional clones. SYLV scion affected RUP stems more than ALF did and interestingly led to a depletion in the *myo*-inositol content and to a simultaneous increase in its vanillic acid content, which is a phenolic acid. At this time, available evidence showed that the bacteria and fungi of cucumber (*C. sativus* L) rhizosphere soil responded differently to vanillic acid leading to a lower increase in fungi abundance than in the bacterial one [53]. Furthermore, the soil microbes and the root exudates of grapevines were affected when treated with 4-hydroxybenzoic acids [54]. These findings might be related to the fact that *V. vinifera* subspecies *sylvestris* is known to present a higher tolerance towards downy and powdery mildews and black rot pathogens [42]. In this study, although some sugars were depleted in RUP stems in response to a heterologous scion, especially with SYLV, other sugar compounds were also enhanced suggesting a more balanced carbon metabolism in the graft combinations with RUP rootstocks than the ones with 110R. Indeed, while the scion phloem exudate was barely affected by a heterologous rootstock, except for SY383, all grafts composed of 110R rootstock showed a reduction in sucrose in the phloem harvested below the union, which alerts for a possible unpaired graft union translocation in *V. vinifera* scions grafted onto 110R.

### 3.3. Phloem Exudate Composition Appears Significantly Altered between Scion and Rootstock

By profiling the metabolome of scion and rootstock samples, 27% of the phloem exudate metabolome (i.e., 21 on 78 metabolites) was consistently found to differ between scion and rootstock and 40% of the stem metabolome (i.e., 111 on 277 metabolites) was consistently changed between the two analyzed groups. Taking into consideration that phloem composition is not expected to vary much within the same plant species, it is astonishing that almost one-third of the phloem exudate metabolome is altered between scion and rootstock samples within the same grafted plant. Nevertheless, it was recently shown that the metabolic composition of grafted *Citrus*’s phloem content was affected by rootstock–scion interactions [17]. Specifically, it seems that the degree of interaction in the rootstock phloem sap of *Citrus* is greater than the metabolites affected in the scion phloem sap. Furthermore, sucrose and GABA were highlighted among the phloem metabolites affected by both scion and rootstock [17]. We have shown that sucrose was significantly depleted in the phloem exudate composition collected below the graft union compared to the above union. Nevertheless, given that the sugar concentrations did not appreciably change in Eucalyptus phloem sap (bled from cut bark) collected at different trunk heights (from 0.1 to 3 m) [55], the implication of grafting, rather than distance to the source, seems to be a more probable explanation for the detected sucrose depletion in phloem exudate collected from the rootstock.

In stems, several compounds were enriched in the scion rather than in the rootstock. Among these, carboxylic acids intermediates of the TCA cycle were again enhanced; quinic and shikimic acids involved in phenol metabolism; a number of polyhydroxy acids; phenolic compounds such as caffeic acids and a catechin/epicatechin-like compounds; and sugars and polyols including *myo*-inositol described as discriminative of grapevine pathogen resistance [42]. These results, once again, suggest the presence of a defense reaction in scion stems coupled with the accumulation of sugars above the union. On the contrary, several other phenolic compounds were accumulated in rootstocks, such as resveraltrol (*cis*- and *trans*-) and another compound similar to catechin/epicathechin. *Trans*-resveratrol production was identified in grapevine leaves after pathogen infection and described as a precursor to fungal toxicity compounds identified as phytoalexins [56,57]. Similarly, catechin and epicatechin were also proposed as grapevine graft incompatibility markers [28] and were found accumulated in pathogen-susceptible *V. vinifera* cultivars together with caffeic acid [42]. Interestingly, phenols were not only enhanced in the rootstock, which is expected due to the lignification of its tissue but also enhanced in herbaceous scion stems, suggesting a possible role in plant defense. Aside from that, differences in scion and rootstock tissues (age and lignification) must also be taken into consideration since the tissue was revealed as the highest variance factor in the PCA (Supplementary Figure S1).

In summary, we have shown that in grapevines both grafting partners can exert their influence in specific organs and phloem exudates, according to the specific graft combination. Heterografting seems to affect rootstocks more than scions and we confirmed that both scion and rootstocks perceive the presence of a heterologous grafting partner leading to the induction of defense-related metabolites. This phenomenon is not only restricted to the cells close to the graft interface, as previously proposed [32], but is also detected in distant leaves. We also conclude that leaves are the best choice of tissue to search for grafting-related metabolic markers as they show more consistent changes (Figure 3A). Notably the effect of a scion on a rootstock was genotypically-driven and not generalizable (i.e., different scions lead to different effects on rootstocks). Surprisingly, the phloem exudate composition was significantly altered between the scion and rootstock and sucrose was found specifically depleted in the rootstock phloem exudate in several *V. vinifera* scions when grafted onto 110R rootstock suggesting an impaired translocation across the graft union of these grafts. Taking into consideration that the phloem is the main route for the exchange of photoassimilates and signals between grafting partners, more studies on the phloem content seem to be necessary to elucidate the grapevine scion–rootstock interactions.

## 4. Materials and Methods

### 4.1. Experimental Design and Plant Material

The experimental design comprised of three American rootstocks: Richter-110 (*V. berlandieri* × *V. rupestris*, 110R, JBP/PT clone), *V. rupestris* (RUP), and *V. berlandieri* (BERL); and of six *V. vinifera cultivars*: Syrah clone 383 and 470 (SY383 and SY470, ENTAV-INRA/FR clones), Touriga Nacional clone 21 and 112 (TN21 and TN112, ISA/PT and JBP/PT clones, respectively), and Alfrocheiro (ALF) and *V. vinifera* subsp. *Sylvestris* (SYLV). Certified virus-free cuttings of TN21, TN112, SY470, and 110R were supplied by the Plansel nursery in Montemor-o-Novo, Portugal (291 m above sea level, 38°39′ N, and 8°13′ W). The remaining plants were collected from the Portuguese National Ampelografic Collection (PRT051), located at Quinta da Almoinha, Dois Portos, Torres Vedras, Portugal (39°02′34.03″ N, −9°10′57.41″ W). The following heterograft combinations, as well as their respective homografts, were performed at the end of April 2018: TN21/110R, TN112/110R, SY383/110R, SY470/110R, ALF/RUP, SYLV/RUP, ALF/BER, and SYLV/BERL. One hundred biological replicates per graft combination were made, except for the grafts with *V. berlandieri* rootstock for which only 20 replicates per combination were available. All grafts were made under commercial nursery conditions by the bench omega-grafting method using dormant cuttings. The grafts were stratified for 21 days to induce callus formation at the graft zone [33], plotted in pots (510 cm^3^ volume), and grown under greenhouse conditions with average day and night temperatures of 20 °C and 23 °C, respectively, and relative humidity of 68 % and 75%, in Oeiras, Portugal, for hardening and to minimize environmental interferences. Supplementary Table S1 summarizes the analyzed graft combinations.

### 4.2. Sample Collection

**Figure 6:**
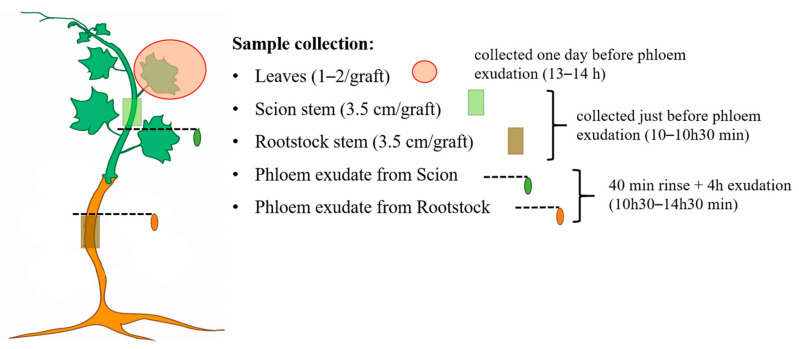
Sample collection scheme. Per graft combination one to two leaves, a segment of 3.5 cm of the scion stem, and 4 h of phloem exudate were collected from scions at 5–6 months after grafting. Rootstock stem samples were harvested from stem segment (length of 3.5 cm) and phloem exudate were collected for 4 h. Leaves were collected one day before phloem exudation to permit the plant to recover. Each sample type was collected at the same circadian phase.

Samples were collected according to the formation of 10–12 nodes on grafted scions 5–6 months after grafting. Each sample is a pool of 5 grafted plants, scion leaves (1–2 expanded leaves/graft), scion’s and rootstock’s stem (10–15 cm above and below the graft union, respectively), and phloem exudate from both scion and rootstock sources (15–20 cm above and below the graft union, respectively) were collected as indicated in Figure 6.

Phloem exudate (five biological replicates per sample) were collected from scion (with 5–6 leaves) and rootstock (with at least 2 scion’s healthy leaves) stems cut under EDTA (10 mM EDTA, pH 7.5) solution and submerged in Falcon tubes containing 10 mL EDTA (for scions) and 20 mL EDTA (for rootstocks). The first 40 min of exudate was discarded to avoid contaminations from cut-derived cellular debris. The base of the stems was then submerged under a new EDTA solution and placed on a closed plastic bag filled with water to avoid plant transpiration to facilitate the collection of phloem sap. After 4 h of exudation, the plant material was discarded and the EDTA-phloem sap sample centrifuged (for 5 min at 3400 rcf). Of the supernatant, 10% aliquots (1 mL for scion’s phloem exudate and 2 mL for the rootstock) were frozen in liquid nitrogen, and stored at −80 °C until further analysis. The quality of the scion phloem exudate was previously assessed in EDTA and non-EDTA (water) control samples by monitoring the sugar composition (i.e., sucrose, glucose, and fructose) in the exudate every two hours of collection (up to 6 h) by 1D Proton NMR analysis (data not shown).

### 4.3. GC-MS Metabolite Profiling of Leaves, Dtems, and Phloem Exudate

Polar metabolite samples were extracted from 85 mg ± 10 mg fresh weight of ground leaves and stem segments as described by Erban et al. (2020) [58]. Briefly, 300 µL of 100% pre-cooled methanol (MeOH), 30 µL of nonadecanoic acid methylester (2 mg/mL stock in CHCl_3_), and 30 µL of 0.2 mg/mL U-^13^C-sorbitol in MeOH were added to each sample and mixed for 15 min at 70 °C. The amount of 200 µL of CHCl_3_ was added and mixed for 5 min at 37 °C. Afterwards, 400 µL of double distilled H_2_O was added. The resulting mixture was shaken and centrifuged (for 5 min at 20,800 rcf) to separate predominantly polar and non-polar liquid phases. From the upper polar phase, aliquots of 160 µL were each collected and dried in a Speed Vacuum concentrator overnight. Dry samples were stored at −20 °C. Phloem exudates, namely 1 mL of scion exudate or 2 mL of rootstock exudate were freeze-dried and omitted the extraction procedure. Derivatization of freeze-dried phloem samples and predominantly polar leaf or stem extracts was carried out by methoxyamination and trimethylsilylation [58]. An n-alkane mixture was used to determine retention time indices [58]. Briefly, 40 µL of methoxyamine hydrochloride in pyridine and 20 mg/mL were added to each sample and mixed for 90 min at 30 °C. Afterwards, 80 µL BSTFA-mix, i.e., 70 µL BSTFA plus 10 µL n-alkane-mixture were added and incubated 30 min at 37 °C. The amount of 1 µL of derivatized-sample was analyzed both by 1:30 volume ratio split-injection and by splitless injection modes using a gas chromatography–electron impact ionization-time of flight/mass spectrometry (GC–EI–TOF/MS) instrument. Instrument and instrument settings were as described previously [58]. ChromaTOF software was used for data acquisition and baseline correction. Processing of chromatography data and peak annotation was carried out using the TagFinder visualization and pre-processing tool [59]. Substance annotation was manually supervised by comparison of retention time indices and mass spectra of reference metabolites from the Golm Metabolome Database, http://gmd.mpimp-golm.mpg.de/, accessed on 26 April 2021 [60]. Metabolite annotations by mass spectral and retention index match are considered verified. Other annotations were by mass spectral match using the AMDIS build 121.86 and MSSearch version 2.0f software (https://chemdata.nist.gov/mass-spc/ms-search/, accessed on 26 April 2021). These annotations are indicated by the prefix “similar to” following the chemical class or the best matching compound [61]. Metabolite names reflect the current identification status of compound or compound class, respectively.

### 4.4. Comprehensive Non-Targeted and Targeted Data Analysis of GC-MS Profiles

We performed non-targeted data analysis in combination with targeted analyses of metabolites that were represented by the subset of annotated mass features [59,62]. Non-targeted data analysis of all mass features monitored by split and splitless GC-EI-TOF/MS metabolite profiling modes ensured comprehensiveness and included unexpected metabolites and metabolic changes of the predominantly polar metabolite fractions from leaf and stem material or phloem exudates.

Stems and leaves datasets were baseline-corrected responses, i.e., arbitrary abundances of chromatographic peak heights of recorded mass-features. These responses were normalized to the response of the U^13^-sorbitol internal standard and fresh weight after chemical background subtraction using mean responses of non-sample controls. Non-sample controls (n = 4 per subset) were empty samples prepared at the metabolite extraction step and carried throughout the entire analytical procedure. The phloem exudate datasets were identically processed but lacked internal standardization and non-sample controls. These data were normalized to the sum of responses of selected analytes (Supplementary Table S2, spreadsheet “phloem”, cells: KG41-KG56 and KG99-KG101).

For statistical analysis, background corrected and normalized data were divided by the median across all samples per mass feature and log_10_-transformed. Statistical analyses were executed by the R statistical programming software, R version 3.6.2 (www.r-project.org, accessed on 26 April 2021) and RStudio version 1.2.5033 (http://www.rstudio.com/, accessed on 26 April 2021) using the MetaboAnalyst R package v2.0.1 [63]. Data integrity check with default parameters of the package and inter-quantile range filtering was performed followed by one-way ANOVA and Tukey post hoc tests, including FDR-correction of the ‘p.adjust’ R-function (https://www.rdocumentation.org/packages/stats/versions/3.6.2/topics/p.adjust, accessed on 26 April 2021) as the integral part of the MetaboAnalystRv2.0.0 package. The significance threshold was *p* < 0.05. Significantly changed mass features were retrieved from the Tukey multiple-comparison tables. Only those mass features that we recorded in at least 75% of the replicate sets and the mass feature that were simultaneously present in >75% of the replicates of a graft combination and <25% of the replicates of another graft combination were considered. Spurious recordings were omitted from further analyses. In the case of homografted vs. heterografted plants and paired, i.e., graft combination, comparisons of the phloem and stem datasets, the ANOVA and Tukey test were carried out separately for scion and rootstock samples using independently normalized and transformed data subsets.

Principal component analyses (PCAs) were computed using the log_10_-transformed data sets. PCA was performed by the MetaboAnalyst R package. Heat maps were generated to analyze relevant differences between metabolic profiles of homografts and heterografts and of the scion comparison to rootstock by applying the ComplexHeatmap R package [64] to a selection of significantly changed metabolites. Specifically, only those metabolites that differentially accumulated significantly and consistently across the diverse graft combinations per group were included. The consistency criterion was an occurrence in at least 80% of the graft combinations per group. Log_10_-transformed ratios compared to the metabolite means per graft combination were visualized.

Presented results from analyses of paired graft combinations are mean values ± standard error (SE) of data that were maximally normalized. Significant differences are reported at three threshold levels, namely * *p* < 0.05, ** *p* < 0.01, *** *p* < 0.001.

## Data Availability

The data presented in this study are available in article.

## Supplementary Materials

The following are available online at https://www.mdpi.com/article/10.3390/metabo11060349/s1. Figure S1: Scores plot between the first two components of each separate PCA: (a) in leaves; (b) phloem exudate; and (c) stems datasets for all analyzed samples. Table S1: Graft combinations and plant species analyzed and their known compatibility behavior. Table S2: Excel file of raw data, statistic results, and experimental details.
