# Supplementary material for: The Impact of Metabolic Scion–Rootstock Interactions in Different Grapevine Tissues and Phloem Exudates
Source: Metabolites. 2021 May 30;11(6):349. doi: 10.3390/metabo11060349 (PMC8228596; doi:10.3390/metabo11060349)
Supplement: Supplementary file 1 [file metabolites-11-00349-s001.zip › metabolites-1217694-supplementary.pdf]

Supplementary Materials: The following are available online at www.mdpi.com/xxx/s1, Figure S1: Scores plot between the first two components of each separate PCA: (**a**) in leaves; (**b**) phloem exudate; and (**c**) stems datasets for all analyzed samples, Table S1: Number of biological replicates and samples analyzed per graft combination, Table S2: Excel file of raw data, statistic results, and experimental details.

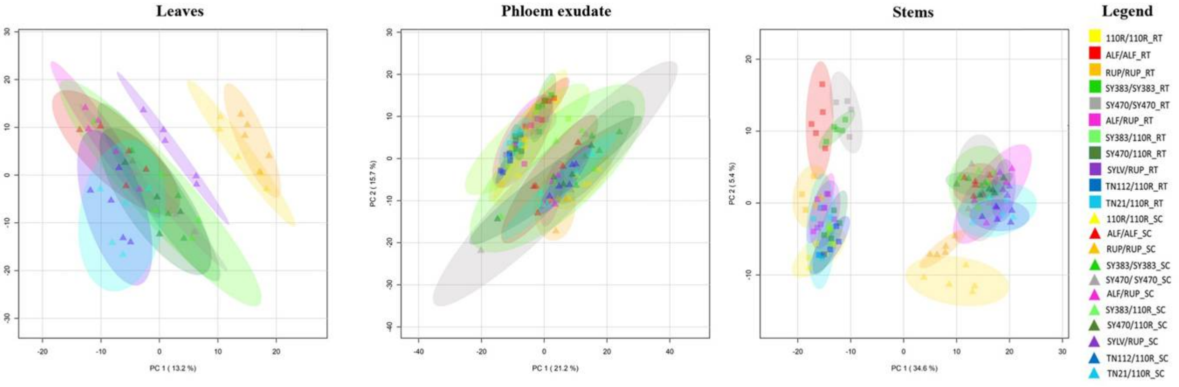

(**a**) (**b**) (**c**)

Figure S1. Scores plot between the first two components of each separate PCA: (**a**) in leaves; (**b**) phloem exudate; and (**c**) stems datasets for all analyzed samples. The variances are shown in brackets and the ellipses show 95% confidence intervals. The square symbol indicates samples collected from the rootstock and the triangle samples collected from the scion. Different colors discriminate the graft combination analyzed. (n = 5 per graft combination, except for SY470 homografts for which n = 4).

Table S1. Number of biological replicates and samples analyzed per graft combination.

Graft combination	Scion species	Rootstock species	Graft type
**110R/110R**	(*V. berlandieri* x *V. rupestris*)	(*V. berlandieri* x *V. rupestris*)	Homograft
**ALF/ALF**	*V. vinifera* cv. Alfrocheiro	*V. vinifera* cv. Alfrocheiro	Homograft
**RUP/RUP**	*V. rupestris Du Lot*	*V. rupestris Du Lot*	Homograft
**SY383/SY383**	*V. vinifera* cv. Syrah, clone 383	*V. vinifera* cv. Syrah, clone 383	Homograft
**SY470/SY470**	*V. vinifera* cv. Syrah, clone 470	*V. vinifera* cv. Syrah, clone 470	Homograft
**ALF/RUP**	*V. vinifera* cv. Alfrocheiro	*V. rupestris Du Lot*	Heterograft
**SYLV/RUP**	*V. vinifera* subsp. *Sylvestris*	*V. rupestris Du Lot*	Heterograft
**SY383/110R**	*V. vinifera* cv. Syrah, clone 383	(*V. berlandieri* x *V. rupestris*)	Heterograft
**SY470/110R**	*V. vinifera* cv. Syrah, clone 470	(*V. berlandieri* x *V. rupestris*)	Heterograft
**TN112/110R**	*V. vinifera* cv. Touriga Nacional, clone 112	(*V. berlandieri* x *V. rupestris*)	Heterograft
**TN21/110R**	*V. vinifera* cv. Touriga Nacional, clone 21	(*V. berlandieri* x *V. rupestris*)	Heterograft

Table S2. Excel file of raw data, statistic results, and experimental details. Leaves, phloem exudate, and stems datasets are contained in separated spreadsheets. Available at: https://drive.google.com/file/d/1efyrzJT4j1augdrCABvsUI84bbNsMlQY/view?usp=sharing
